# Caffeine and human performance: from molecular mechanisms to exercise and recovery

**DOI:** 10.3389/fphys.2026.1871149

**Published:** 2026-07-09

**Authors:** Jingzhi Leng, Jingjing Yu, Zheheng Li, Yimin Zhang, Juntao Cui

**Affiliations:** 1 College of Sports and Human Science, Key Laboratory of Sports and Physical Fitness Health of Ministry of Education, Beijing Sport University, Beijing, China; 2 Department of Gastroenterology, Xinhua Hospital, Shanghai Jiaotong University, School of Medicine, Shanghai, China; 3 School of Physical Education, Qingdao University, Qingdao, China; 4 Institute of Brain Science and Disease, Shandong Provincial Collaborative Innovation Center for Neurodegenerative Disorders, Shandong Key Laboratory of Pathogenesis and Prevention of Brain Diseases, Qingdao University, Qingdao, China

**Keywords:** adenosine receptor antagonist, caffeine, energy metabolism, exercise performance, sport nutrition

## Abstract

Caffeine, the world’s most widely consumed psychoactive substance, is a well-established ergogenic aid. Its performance-enhancing effects are mediated through multiple, interacting mechanisms that follow a dose-dependent hierarchy. The primary and most significant action at common dietary doses is the antagonism of central adenosine A_1_ and A_2a_ receptors. This central blockade reduces perceived effort and fatigue, increases arousal, and modulates pain perception, thereby lowering the neural cost of exercise. This central effect is complemented by secondary peripheral metabolic actions, such as the promotion of lipolysis, though their role as primary drivers in exercising humans is supported mainly by experimental models. A direct effect on skeletal muscle calcium handling occurs only at supra-physiological concentrations and is not relevant in practice. The integration of these mechanisms leads to significant but task-dependent performance outcomes. The most robust and consistent benefits are observed for aerobic endurance exercise. Effects on strength, power, and high-intensity intermittent performance are more variable and are largely mediated by neurological and perceptual factors, explaining their greater consistency in protocols involving fatigue. For sports requiring cognitive skill, caffeine reliably enhances reaction time, vigilance, and accuracy via central nervous system stimulation. The ergogenic response is highly individualized, shaped by a complex interplay of dose, timing, genetics, and habitual use. Furthermore, preliminary evidence suggests caffeine may modulate post-exercise recovery by potentially accelerating muscle glycogen resynthesis when co-ingested with carbohydrates and by improving muscle perfusion. However, leveraging any potential recovery benefit necessitates extremely careful timing to avoid sleep disruption, as protecting sleep quality is a paramount recovery priority. Future research should develop sport- and context-specific protocols, clarify long-term adaptations, and advance personalized strategies that integrate genetic, habitual, and temporal factors to maximize benefits and minimize adverse effects, reinforcing caffeine’s role as a cornerstone of evidence-based sports nutrition.

## 1 Introduction

Caffeine, a naturally occurring methylxanthine alkaloid found in coffee beans, tea leaves, cacao, and other plants, has profoundly evolved from a simple dietary staple into a pervasive socio-cultural phenomenon deeply embedded in daily rituals and global economies (Poole et al., 2017; Nehlig, 2018; Schuster and Mitchell, 2019). It holds the distinction of being the most widely consumed psychoactive substance worldwide, with its use spanning centuries and cultures. Beyond its role in promoting wakefulness, caffeine has been rigorously investigated and is now firmly established as a safe and effective ergogenic aid, endorsed and strategically utilized across diverse athletic disciplines, from elite competitive sports to recreational fitness communities (Cappelletti et al., 2015; McLellan et al., 2016). A substantial and robust body of research consistently confirms that acute caffeine ingestion can significantly enhance central alertness, improve cognitive mood states, and, most notably for athletes, reduce the perception of effort during physical activity (Barcelos et al., 2020; Meamar et al., 2024). These neuro-modulatory effects ultimately translate into measurable, practical improvements in physical performance. The ergogenic benefits are well-documented across various exercise modalities, it can extend time to exhaustion in prolonged submaximal endurance activities, increase mean power output in cycling time trials, and enhance performance in high-intensity intermittent exercise paradigms that mimic the demands of team sports (Kasper et al., 2016; Glaister and Gissane, 2018; Chen et al., 2024).

The physiological basis for these ergogenic effects, however, is complex and extends beyond a single mechanism. Early research, focusing on endurance performance, often attributed caffeine’s benefits primarily to a peripheral metabolic mechanism, the promotion of lipolysis and fat oxidation, thereby potentially sparing muscle glycogen (Laurent et al., 2000; Graham, 2001). While this metabolic shift contributes, contemporary understanding frames it as one component within a broader, multi-system interaction. The primary and most significant action of caffeine at common dietary doses is the antagonism of adenosine receptors in the central nervous system (CNS), which underlies its effects on vigilance, motivation, and pain perception (Costenla et al., 2010; Astorino et al., 2011; Nehlig, 2016). Concurrently, through secondary and dose-dependent pathways, caffeine can influence peripheral processes related to energy metabolism and muscular function. The synergistic interplay between these central and peripheral actions underpins its broad-spectrum efficacy as a performance enhancer (Nehlig, 2018; Guest et al., 2021).

Given the extensive existing literature on caffeine and exercise, this narrative review aims to provide a unique synthesis by proposing and applying a dose-dependent hierarchical and integrative framework. This framework positions central adenosine receptor antagonism as the indispensable primary mechanism at common intake levels, with peripheral metabolic actions as secondary supports. We will first delineate these key molecular and cellular mechanisms, critically distinguishing between those strongly supported by human exercise studies and those derived primarily from experimental models. Subsequently, we will examine how this mechanistic hierarchy integrates to differentially influence physiological function and subjective perception across various exercise domains. The translation of these integrated effects into performance outcomes will be critically evaluated, with a focused analysis on the sources of heterogeneity in areas like strength and power performance. Furthermore, we will provide a balanced and cautious discussion on the potential, yet complex, role of caffeine in post-exercise recovery, explicitly weighing preliminary mechanistic benefits against the well-established risk of sleep disruption. Finally, the review will synthesize the critical moderators of inter-individual variability including genetics, habituation, and the placebo effect to advance towards a more personalized application perspective. By integrating this updated mechanistic framework with applied performance, recovery, and individual variability considerations, this review aims to offer a coherent and evidence-informed guide for optimizing caffeine use in athletic contexts.

## 2 Methods

This work is a comprehensive narrative review that synthesizes and critically evaluates the current evidence on the roles of caffeine in energy metabolism, exercise performance, and post-exercise recovery. Its primary objective is to integrate findings from molecular, physiological, and applied perspectives into a coherent explanatory framework. Relevant literature was identified through systematic electronic searches conducted in PubMed/MEDLINE, Web of Science, and Google Scholar databases, with the final search completed in May 2026 to encompass the evidence published up to that point. The search strategy employed combinations of keywords and Boolean operators, using the core search string (caffeine OR coffee) AND (exercise performance OR ergogenic aid OR sports nutrition OR adenosine receptor OR central nervous system OR energy metabolism OR lipolysis OR glycogen OR recovery OR muscle glycogen resynthesis OR sleep OR dose-response OR individual variability OR genetics). This approach aimed to capture both seminal works and recent evidence, with particular emphasis on high-impact reviews, meta-analyses, and original research published in peer-reviewed journals.

The literature selection followed a structured, iterative, and concept-driven process to ensure a comprehensive yet focused synthesis. This process, conducted primarily by the lead author with critical discussion and validation by all co-authors, involved several key stages. First, titles and abstracts of records retrieved from the database searches were screened for relevance to the review’s core themes, which include caffeine’s mechanisms of action, its effects across different exercise modalities, and its role in post-exercise recovery. The full texts of articles deemed potentially relevant from this initial screen were then retrieved and evaluated in depth. To ensure the inclusion of seminal and recent key studies, the reference lists of identified high-impact reviews, meta-analyses, and original articles were examined in a backward snowballing approach. Furthermore, targeted searches were conducted for literature addressing specific contradictions or emerging topics highlighted during the ongoing synthesis phase. During the evaluation, priority was given to original human intervention studies on caffeine supplementation related to exercise performance or recovery, to mechanistic studies elucidating caffeine’s actions on adenosine receptors, metabolism, or neuromuscular function, and to systematic reviews and meta-analyses in relevant sub-fields. Articles discussing critical moderators of caffeine’s effects were also included. Studies were excluded if they were non-English publications, featured significant methodological limitations as noted in peer critiques or subsequent commentary, or focused solely on the general health effects of caffeine unrelated to exercise physiology. This multi-stage process ensured that the final narrative synthesis was informed by a robust, relevant, and critically appraised body of literature, forming a transparent foundation for the review.

## 3 Molecular and cellular mechanisms of action

**Figure 1:**
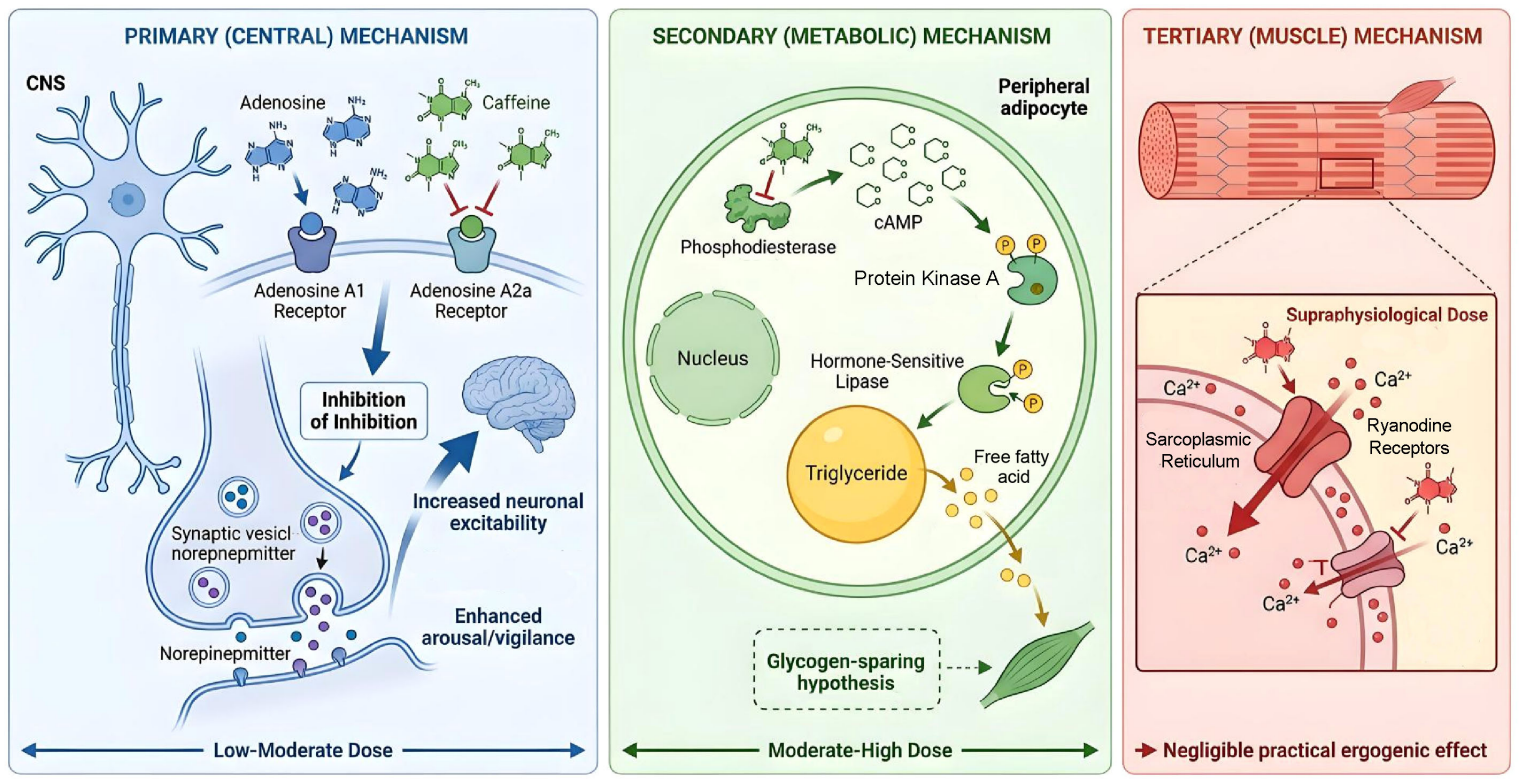
The dose-dependent, multi-pathway mechanisms of caffeine action. Caffeine exerts its ergogenic effects primarily by antagonizing central adenosine A_1_ and A_2a_ receptors at common intake levels, reducing perceived effort and increasing alertness. At higher doses, it may also inhibit phosphodiesterase to promote lipolysis, providing a metabolic basis for glycogen sparing, though this is supported mainly by experimental models. A direct effect on skeletal muscle calcium release occurs only at supraphysiological doses and is negligible in practice.

The ergogenic effects of caffeine are not attributable to a single action but result from the interplay of multiple molecular pathways, the relative contribution of which is critically dose-dependent​ (**Figure 1**). At physiologically relevant concentrations achieved through typical dietary or supplemental intake, the primary and most significant mechanism​ is the antagonism of central adenosine receptors. Putative secondary metabolic effects, such as enhanced lipolysis, may occur but are mediated through pathways like phosphodiesterase inhibition whose *in vivo* relevance at common ergogenic doses remains less certain and is supported largely by experimental models. In contrast, direct effects on skeletal muscle calcium handling​ are observed only at supraphysiological concentrations unattainable with safe consumption and are therefore not considered relevant for practical ergogenic use. This dose-dependent hierarchy of mechanisms from central nervous system modulation as the cornerstone, to peripheral metabolic support, and finally to experimentally isolated muscular effects provides the foundational framework for understanding caffeine’s actions in exercise physiology.

### 3.1 Primary mechanism: central adenosine receptor antagonism

The foremost mechanism underlying caffeine’s ergogenic effects is its action as a non-selective competitive antagonist of adenosine receptors, primarily the A_1_ and A_2a_ subtypes (Ribeiro and Sebastião, 2010; Nehlig, 2018). By structurally mimicking adenosine, caffeine effectively blocks its binding. This action counteracts adenosine’s generally inhibitory neuromodulatory tone, which under normal conditions promotes sleepiness and suppresses arousal (Nagata and Urade, 2012; Turnbull et al., 2017; Reichert et al., 2022). Within the central nervous system, this blockade produces region-specific consequences. Antagonism of A_1_ receptors in areas such as the cortex and hippocampus increase neuronal excitability. Conversely, inhibition of striatal A_2a_ receptors potentiates dopaminergic and glutamatergic neurotransmission (Li et al., 2011; Ferré et al., 2023). The resultant neurochemical shift, characterized by elevated synaptic levels of neurotransmitters like dopamine and norepinephrine, is the foundation for the well-documented subjective experiences of increased alertness, improved mood, and enhanced vigilance (Volkow et al., 2015). This central mechanism is unequivocally established as the primary driver of caffeine’s effects on perception of effort, fatigue, and cognitive performance at typical dietary and supplemental doses.

### 3.2 Secondary mechanism: metabolic effects via phosphodiesterase inhibition

A secondary proposed mechanism involves the non-selective inhibition of phosphodiesterase enzymes, which degrade cyclic adenosine monophosphate (Pohanka, 2015; Faudone et al., 2021). In experimental models, this inhibition elevates intracellular cAMP, activating protein kinase A, which may then phosphorylate targets such as hormone-sensitive lipase to promote lipolysis (Guest et al., 2021; Kudo et al., 2024). This cascade provides a biochemical basis for the historical glycogen-sparing hypothesis, wherein increased fat oxidation could theoretically preserve muscle glycogen (Costill et al., 1978). It is critical to note, however, that robust evidence for this specific mechanism as a primary driver in exercising humans at typical ergogenic doses is limited and derives largely from *in vitro* or high-dose pharmacological models. Its *in vivo* significance and contribution during exercise remain debated. The current consensus posits phosphodiesterase inhibition and the associated metabolic shifts as a secondary, supportive pathway that may amplify other signals rather than act as a primary ergogenic driver during exercise in humans.

### 3.3 Tertiary mechanism: direct muscle calcium handling

At supraphysiological concentrations caffeine can directly influence skeletal muscle excitation-contraction coupling (Figueroa et al., 2019). This effect is mechanistically distinct from its receptor-mediated actions. In experimental settings, caffeine is proposed to sensitize ryanodine receptors on the sarcoplasmic reticulum, thereby promoting greater Ca²^+^ release into the cytosol. It may also weakly inhibit Ca²^+^ reuptake, prolonging the elevation of cytosolic Ca²^+^ (Reggiani, 2021; Fukutani et al., 2022). Theoretically, these actions could potentiate muscle contraction. However, the concentrations required to elicit this direct effect are orders of magnitude higher than the peak plasma levels achieved with even high ergogenic doses consumed by athletes. Therefore, while this mechanism is of significant interest in basic muscle physiology and pharmacological research, its practical contribution to the ergogenic effects of caffeine in real-world athletic contexts is considered negligible. This distinction reinforces the conclusion that caffeine’s performance-enhancing properties in practice are mediated primarily through central nervous system actions, not via direct effects on the contractile machinery of skeletal muscle.

## 4 Impact on exercise performance and sport-specific outcomes

The integrated, dose-dependent physiological mechanisms of caffeine translate into measurable improvements in athletic performance. However, the magnitude and consistency of its ergogenic effects are highly dependent on the specific demands of the exercise task, a relationship directly governed by the underlying mechanistic hierarchy. The most robust and well-established benefits are observed for endurance exercise, where the synergy between the primary central nervous system effect and secondary metabolic actions is most clearly realized. In contrast, the evidence for improvements in strength, power, and anaerobic activities is more variable; the benefits that do occur in this domain are mediated largely by neurological and perceptual modulation rather than by direct peripheral metabolic changes, explaining their context-dependency. Furthermore, caffeine’s ergogenic value is distinctly evident and often primary in sports requiring high levels of cognitive skill, where its central stimulant effects directly enhance reaction time, vigilance, and motor accuracy.

### 4.1 Aerobic endurance performance

The most consistent and well-documented performance benefits of caffeine occur during prolonged, moderate-to-high intensity aerobic exercise (Stadheim et al., 2021; Wang et al., 2022). This efficacy is fundamentally attributed to the primary mechanism of central adenosine receptor antagonism, which robustly reduces the perception of effort and fatigue. This central effect is synergistic with potential peripheral metabolic consequences. While the promotion of lipolysis and fat oxidation is often discussed, contemporary evidence from human studies positions it as a secondary or supportive factor rather than a primary driver (Guest et al., 2021). Therefore, the enhancement of endurance performance is best explained by a clear hierarchy of mechanisms, the reduction in neural strain and perceived exertion via central adenosine receptor blockade is the principal, well-supported effect. The potential for increased fat oxidation and substrate availability during prolonged activity (Kreutzer et al., 2022; Saunders et al., 2023) may work in concert as a complementary metabolic action, but its contribution is conditional and less definitively established than the central effect. This integrated, mechanism-driven understanding explains how caffeine enables athletes to sustain a higher power output, maintain pace, and extend time to exhaustion, solidifying its status as a cornerstone nutritional strategy for endurance sports.

### 4.2 Strength, power, and anaerobic performance

The evidence for caffeine’s efficacy in enhancing maximal strength, single-effort power, or pure anaerobic capacity is heterogeneous (San Juan et al., 2019; Schwarz Na and McKinley-Barnard Sk, 2020; Grgic, 2021; Papadakis et al., 2025). While numerous studies report improvements in metrics like one-repetition maximum strength, local muscular endurance, and mean or peak power during repeated efforts, an almost equal number show null or inconsistent effects, particularly for simple, non-fatiguing maximal attempts. The ergogenic benefits that do occur in this domain are likely mediated less by direct peripheral metabolic shifts and more by neurophysiological and perceptual factors, including potential enhancements in neuromuscular drive, motor unit recruitment, and, crucially, reduced perception of pain and effort (Tarnopolsky, 2008; Caldas et al., 2022; Nishikawa et al., 2024). Consequently, the most practical ergogenic effects for strength and power are often observed in performance contexts involving repeated efforts or sustained high-intensity output, such as volume-focused resistance training, high-intensity interval training, or the intermittent explosive efforts characteristic of team sports (Madden et al., 2019; Pakulak et al., 2022; Chen et al., 2025; Martinho et al., 2025).

The inconsistency in findings can be attributed to a complex interplay of methodological and individual factors. Methodologically, divergent outcomes are heavily influenced by the specific exercise modality, the effectiveness of blinding procedures, and the timing of caffeine ingestion relative to the performance test (Beedie, 2010; Del Coso et al., 2026). At the individual level, key physiological moderators introduce significant variability. Pharmacodynamic tolerance from habitual caffeine use can attenuate ergogenic responses, underscoring the importance of washout periods in study design (Pickering and Kiely, 2019; Bezuglov et al., 2025; Carbone et al., 2025). Furthermore, genetic polymorphisms, particularly in the CYP1A2gene, critically affect caffeine metabolism and clearance, creating distinct fast and slow metabolizer phenotypes that contribute to the observed responder/non-responder dichotomy in group studies (Mokkawes and de Visser, 2023; Barreto et al., 2024; Wang et al., 2024). An athlete’s training status and associated neuromuscular adaptations may also modulate the expression of caffeine’s primarily neurological effects in this domain. This critical synthesis suggests that caffeine’s benefits for strength and power are not universal but are contingent on these factors, explaining why more consistent effects are observed in protocols involving fatigue or repeated efforts compared to single, all-out attempts.

### 4.3 Cognitive and skill-based performance

Caffeine’s cognitive-enhancing effects are of primary importance in sports demanding high levels of concentration, rapid decision-making, and fine motor control, such as shooting, archery, e-sports, and racquet sports (Sainz et al., 2020; Pirmohammadi et al., 2023). In these domains, even low-to-moderate doses can significantly improve key performance metrics, including reaction time, visual vigilance, information processing speed, and movement accuracy (Zheng et al., 2014; Moreira-de-Sá et al., 2021; Reich et al., 2024). These benefits are predominantly and directly attributed to caffeine’s central action as an adenosine receptor antagonist. This mechanism increases neuronal excitability and facilitates neurotransmission in brain regions critical for attention, executive function, and psychomotor control (Smith, 2002; Panza et al., 2015; Hall et al., 2025). This represents a distinct, and often primary, dimension of ergogenic support that is mechanistically independent of its peripheral effects on muscle, though the two can synergistically complement each other in sports with combined physical and cognitive demands.

The application of caffeine for cognitive enhancement in sport requires nuanced consideration beyond a simple dose-response relationship. First, the optimal dose may be both sport- and task-specific. Lower doses are often sufficient and preferred for precision sports like shooting or archery, as they maximize alertness and steadiness while minimizing the risk of anxiety or tremor that higher doses might induce (Sainz et al., 2020; Yilmaz et al., 2023). In contrast, fast-paced e-sports or racquet sports might benefit from doses in the moderate range to sustain high-level information processing and reaction time over longer periods (Abian et al., 2015; Pirmohammadi et al., 2023). Second, the significant inter-individual variability discussed in Section 5, driven by genetics and habituation, applies equally here (Rahimi et al., 2024). An athlete’s sensitivity to caffeine’s anxiogenic effects, mediated by ADORA2Agenotype, is a critical factor, as increased anxiety can be detrimental to fine motor skill and decision-making under pressure. Finally, the potent psychological expectancy (placebo) effect must be acknowledged. In skill-based tasks where confidence and focus are paramount, the belief in having ingested caffeine can itself enhance performance, making the disentanglement of pharmacological and psychological effects complex but essential for interpreting study outcomes and personalizing use. Therefore, while the evidence robustly supports caffeine’s cognitive benefits, their practical optimization demands careful attention to dose, individual biology, and the specific cognitive profile of the sport.

## 5 Factors influencing efficacy: dose, timing, and individual variability

**Figure 2:**
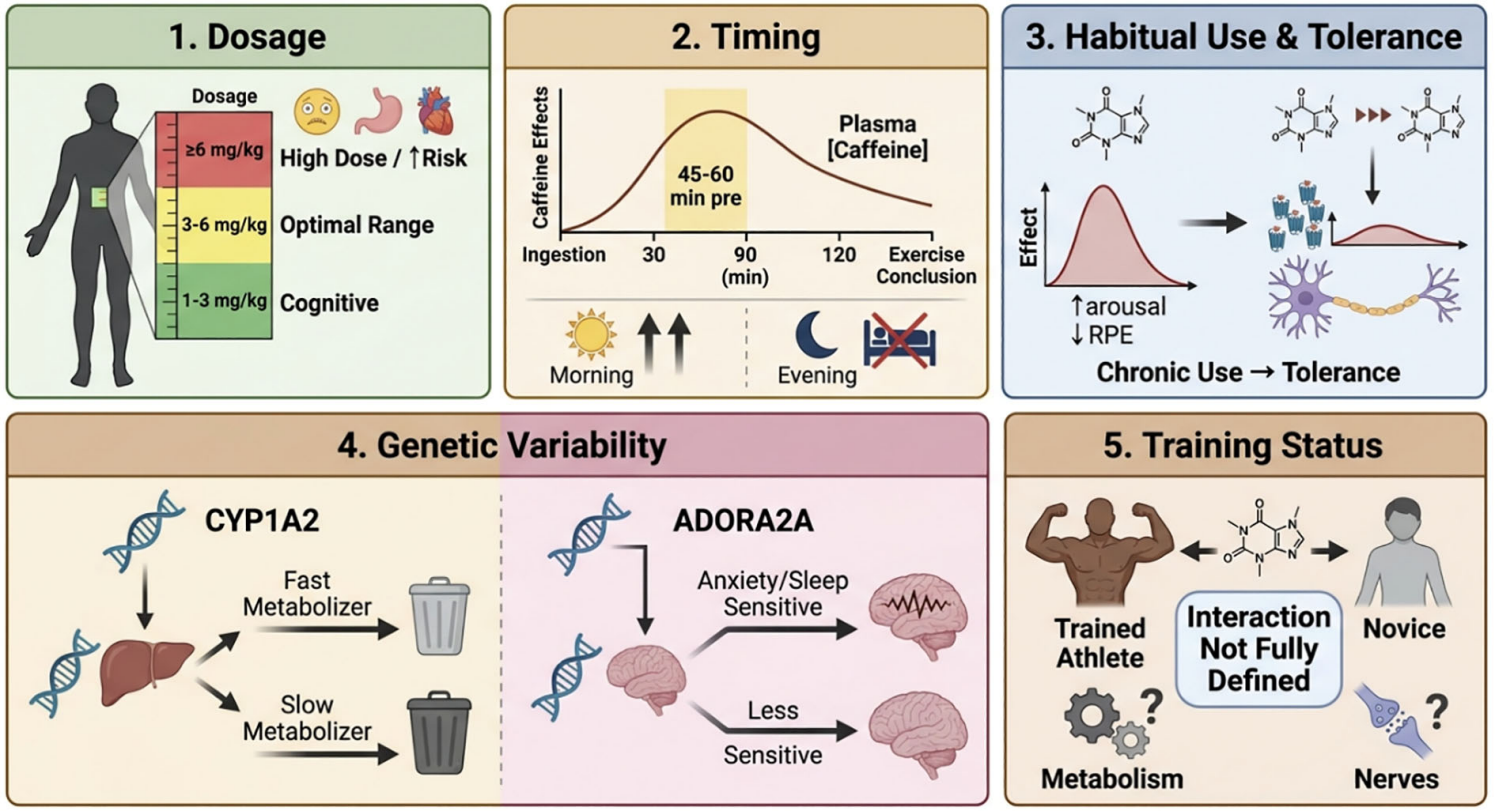
Key factors modulating caffeine’s ergogenic efficacy. The schematic illustrates five primary elements that determine the highly individualized response to caffeine. It covers pharmacokinetic (Dosage & Timing), physiological (Habitual Use/Tolerance & Training Status), and genetic (Genetic Variability) moderators, summarizing their core mechanisms and practical implications for optimizing caffeine use in sports.

The ergogenic response to caffeine is highly individualized, shaped by a complex and dynamic interplay of pharmacological, physiological, and genetic factors (**Figure 2**). This variability means that a standard dose and timing protocol will not produce uniform results across all athletes. Optimizing caffeine’s use, therefore, requires a strategic and personalized approach that carefully considers not only core parameters like dosage​ and timing of ingestion, but also intrinsic moderator variables such as genetic predisposition, habituation and tolerance, and training status. The following sections will critically examine how each of these factors independently and interactively shapes the magnitude and consistency of caffeine’s performance and recovery outcomes.

### 5.1 Dosage

The effective ergogenic dose for most individuals typically ranges from 3 to 6 mg per kilogram of body weight (mg/kg) (Nemati et al., 2023; Antonio et al., 2024). This range, however, encapsulates significant inter-individual variability and context-dependence. Lower doses (1–3 mg/kg) are often sufficient to elicit cognitive and perceptual benefits, such as increased alertness and reduced perception of effort, with a minimized risk of side effects. In contrast, higher doses (≥6 mg/kg) are sometimes employed in an attempt to maximize effects during prolonged endurance events, but this comes with a proportionally increased risk of adverse reactions including anxiety, gastrointestinal distress, and tachycardia (Moo-Puc et al., 2003; Tunnicliffe et al., 2008; Durkalec-Michalski et al., 2019). The evidence for an optimal dosage to enhance strength and power outcomes is less consistent and more debated. While some studies suggest benefits, doses at or above 6 mg/kg may be necessary to elicit measurable effects in this domain, though the response remains highly variable and is likely more dependent on neurogenic than metabolic factors. This underscores the principle that dosage cannot be considered in isolation from the specific performance goal and individual tolerance.

### 5.2 Timing of ingestion

Peak plasma caffeine concentration generally occurs 30 to 90 minutes post-consumption. Therefore, ingestion approximately 45 to 60 minutes before exercise is a common strategy to align peak bioavailability with the start of activity. The time of day also modulates its effects due to circadian rhythms; morning intake may yield greater performance benefits for certain metrics compared to evening intake, which is more likely to impair sleep latency and architecture, negatively affecting recovery. This interaction necessitates a nuanced, context-specific approach to timing, particularly in complex real-world sporting scenarios. For evening or late-day competitions, the standard pre-exercise timing advice becomes problematic as it risks significant sleep disruption. In such cases, evidence-based strategies include using the lowest effective dose, consuming caffeine much earlier in the day, or considering non-caffeine alertness strategies, always weighing the acute performance benefit against the high probability of impaired subsequent sleep. During congested competition schedules, the focus must shift from single-event optimization to managing cumulative sleep debt. This requires planning caffeine use strategically across the entire competitive block, avoiding it in preliminary rounds, and establishing a strict daily cutoff time for intake to protect sleep between events. In the context of travel fatigue and circadian rhythm disruption, caffeine is a double-edged sword. It can be used judiciously upon arrival at a new time zone to promote alertness during local daylight hours, aiding circadian adaptation. However, misuse, particularly consumption too close to the target bedtime, can severely exacerbate sleep disturbances and hinder the adaptation process.

### 5.3 Habitual use and tolerance

Regular caffeine consumption induces pharmacological tolerance, a process mediated primarily by the upregulation of central adenosine receptors. This adaptive response diminishes the magnitude of both the desired ergogenic effects and the unwanted side effects over time. Consequently, habitual users may experience attenuated performance benefits compared to naïve or infrequent users. To restore sensitivity, a period of abstinence or a strategic increase in dosage prior to a key competition is often practiced, though the optimal duration and efficacy of such “washout” periods can vary and require individual experimentation. The development of tolerance is a critical moderator that must be accounted for in both research design and personal supplementation strategies, as it can fundamentally alter the dose-response relationship.

### 5.4 Genetic variability

Genetic polymorphisms are a major, non-modifiable source of inter-individual variability in response to caffeine. Variations in the CYP1A2gene, which encodes the primary cytochrome P450 enzyme responsible for metabolizing caffeine, determine whether an individual is a fast or slow metabolizer. This genetically determined status critically affects the plasma clearance rate and the duration of caffeine’s action, influencing both the timing and magnitude of its effects (Mokkawes and de Visser, 2023; Barreto et al., 2024; Wang et al., 2024). Furthermore, polymorphisms in the ADORA2Agene, which codes for the adenosine A2A receptor, are strongly associated with differential sensitivity to the anxiogenic and sleep-disrupting effects of caffeine (Low et al., 2024; Rahimi et al., 2024). These genetic differences can significantly influence the optimal personalized dose and the individual’s risk profile for adverse reactions, highlighting the potential for genetic screening to inform more precise and safer supplementation strategies.

### 5.5 Training status

An individual’s training status may further modulate the physiological and performance responses to caffeine, potentially through training-induced adaptations in substrate metabolism, neuroendocrine function, or neuromuscular efficiency. For instance, highly trained endurance athletes with optimized fat oxidation pathways might exhibit a different metabolic interaction with caffeine compared to recreationally active individuals. However, the nature and direction of this interaction are not fully delineated and likely vary across different sports and performance phenotypes. While some evidence suggests trained individuals may experience ergogenic benefits, the findings are not uniform. This ambiguity underscores the need for athletes to engage in personalized experimentation to determine their own response, as the effects observed in untrained or generally active populations may not directly translate to the elite athletic context.

## 6 Caffeine in post-exercise recovery

**Figure 3:**
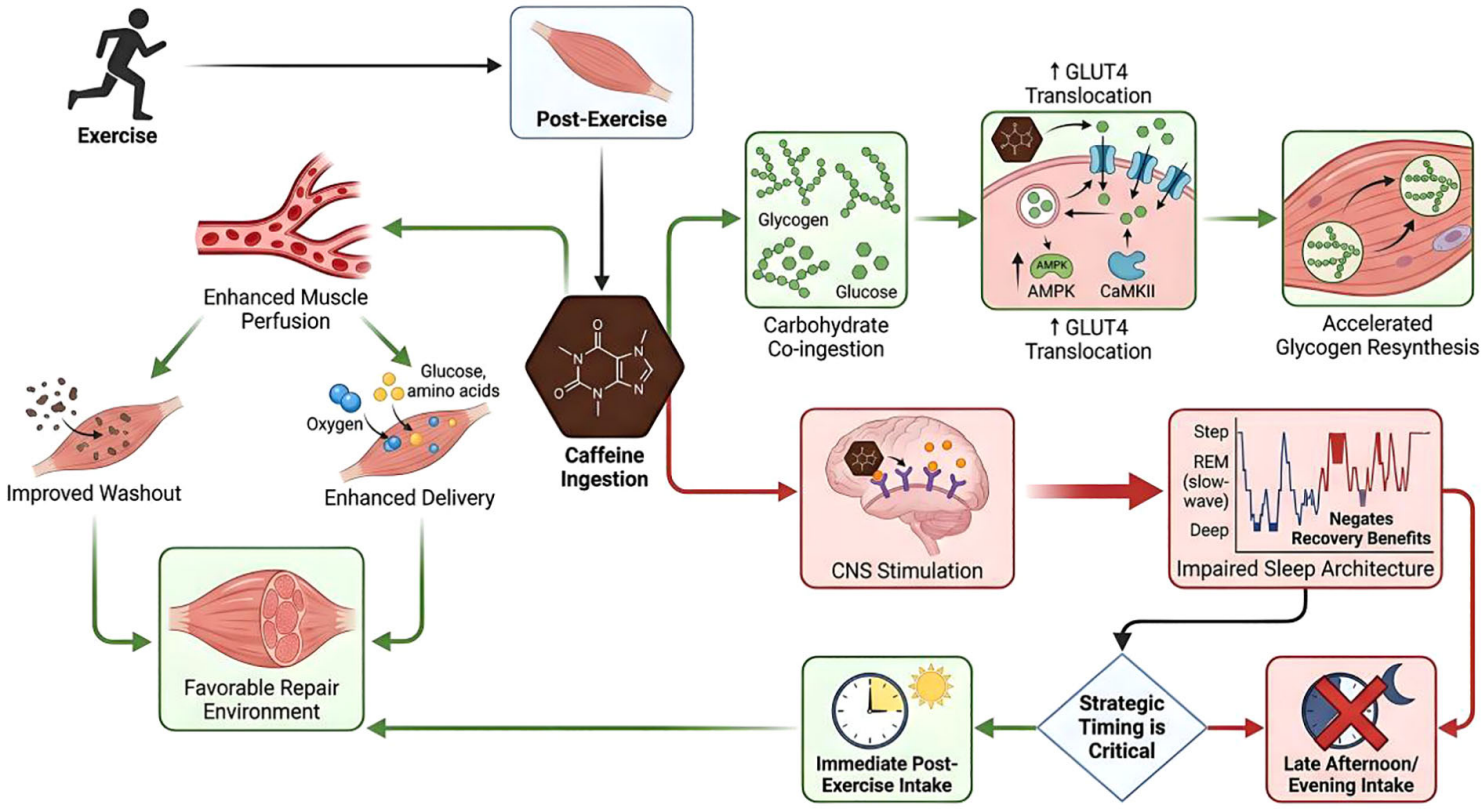
Caffeine’s dual role in post-exercise recovery. This schematic summarizes the dual aspects of caffeine’s role in recovery. It depicts mechanisms for enhancing muscle perfusion to create a favorable repair environment and for accelerating muscle glycogen resynthesis when combined with carbohydrates. A central emphasis is placed on the critical constraint of caffeine’s CNS stimulant effect, which can impair sleep if timed poorly. The figure illustrates the practical need to time intake immediately after exercise while avoiding consumption later in the day.

Beyond its well-established acute ergogenic effects, emerging yet preliminary evidence suggests that caffeine may also modulate selected post-exercise recovery processes, potentially contributing to an athlete’s readiness for subsequent training or competition (**Figure 3**) (Pickering and Grgic, 2019; Loureiro et al., 2021). It is crucial to frame this discussion with caution, as the direct evidence from human recovery studies is more limited and less consistent than the robust literature on performance enhancement. The proposed mechanisms are multi-faceted, targeting both peripheral repair pathways and the restoration of energy substrates; however, their translation into consistent, practical recovery benefits for athletes requires further confirmation and must be carefully weighed against the preeminent risk of sleep disruption.

### 6.1 Enhancement of muscle perfusion and metabolic clearance

A proposed recovery benefit of caffeine may stem from its potential positive effect on skeletal muscle perfusion (Domaszewski et al., 2021; Naderi et al., 2025). Some human studies suggest that post-exercise caffeine ingestion can improve microvascular reactivity and endothelial function, thereby enhancing post-exercise hyperemia and increasing blood flow and oxygen delivery to exercised muscles (Turnbull et al., 2017; Leng et al., 2024). It is proposed that this improved perfusion could facilitate the clearance of metabolic by-products and augment the delivery of oxygen, nutrients, and anabolic hormones. By potentially optimizing this washout and delivery system, caffeine may help correct ionic imbalances, reduce oxidative stress, and support protein turnover, thereby creating a microenvironment that is more favorable for repairing exercise-induced muscle damage and possibly attenuating sensations of stiffness and soreness. However, it is important to note that evidence directly linking these mechanistic observations to consistent, practical improvements in recovery outcomes in athletes is still emerging and requires further confirmation.

### 6.2 Potential synergism with glycogen resynthesis

Another area of interest is caffeine’s reported synergistic effect on muscle glycogen resynthesis when co-ingested with carbohydrates after exercise (Fujii et al., 2025; Naderi et al., 2025). Preliminary evidence indicates that this combination can increase the rate of glycogen replenishment compared to carbohydrates alone, a finding that could be particularly beneficial when recovery time between training sessions or competitions is limited (Loureiro et al., 2021). The proposed cellular mechanism for this effect involves caffeine’s potential to augment the translocation of the glucose transporter GLUT4 to the muscle membrane, possibly via the activation of signaling kinases such as AMPK and CaMKII (Guarino et al., 2013; Mathew et al., 2014; Tsuda et al., 2015; Yun et al., 2024). This could boost insulin-independent glucose uptake, thereby providing more substrate for glycogen synthesis. It is critical to emphasize that this detailed mechanistic evidence derives primarily from *in vitro*and animal models. While the initial human research is promising, it remains more limited and less consistent compared to the extensive literature on caffeine’s acute ergogenic effects. Therefore, while physiologically plausible, the practical application of caffeine for enhancing post-exercise glycogen recovery should currently be considered an emerging strategy based on preliminary evidence.

### 6.3 The critical consideration of sleep disruption

The potent central nervous system stimulant effect of caffeine presents a fundamental consideration and direct challenge to recovery. While it enhances acute alertness, consumption too close to bedtime can significantly impair sleep architecture by prolonging the time taken to fall asleep and reducing sleep quality, including deep and rapid eye movement sleep (Clark and Landolt, 2017; Wang and Bíró, 2021; Gardiner et al., 2023; Chang et al., 2025; Gardiner et al., 2025). As sleep is the most critical component of holistic recovery essential for hormonal regulation, neural repair, and memory consolidation this creates a critical trade-off. Intake that is poorly timed can therefore negate any potential peripheral recovery benefits, making a strategic and individualized approach essential. A practical framework to navigate this distinguishes three primary purposes for caffeine use. The first is for performance, where timing aims to align peak plasma concentration with exercise onset. The second is for recovery, an application where the evidence is more preliminary, if attempted, intake should occur immediately post-exercise to maximize the temporal distance from bedtime. The third and non-negotiable purpose is to maintain a sleep-protection window, enforcing a caffeine-free zone of six to eight hours or more before bedtime while acknowledging individual variation in caffeine metabolism. Ultimately, for any athlete, protecting sleep quality and duration represents a higher-order recovery intervention than any potential marginal gain from post-exercise caffeine. Consequently, the decision to use caffeine, especially in a recovery context, must be individualized, rigorously weighing the uncertain potential for accelerated short-term physiological markers against the fundamental priority of safeguarding sleep-dependent, long-term recovery.

## 7 Critical perspectives and emerging considerations

**Figure 4:**
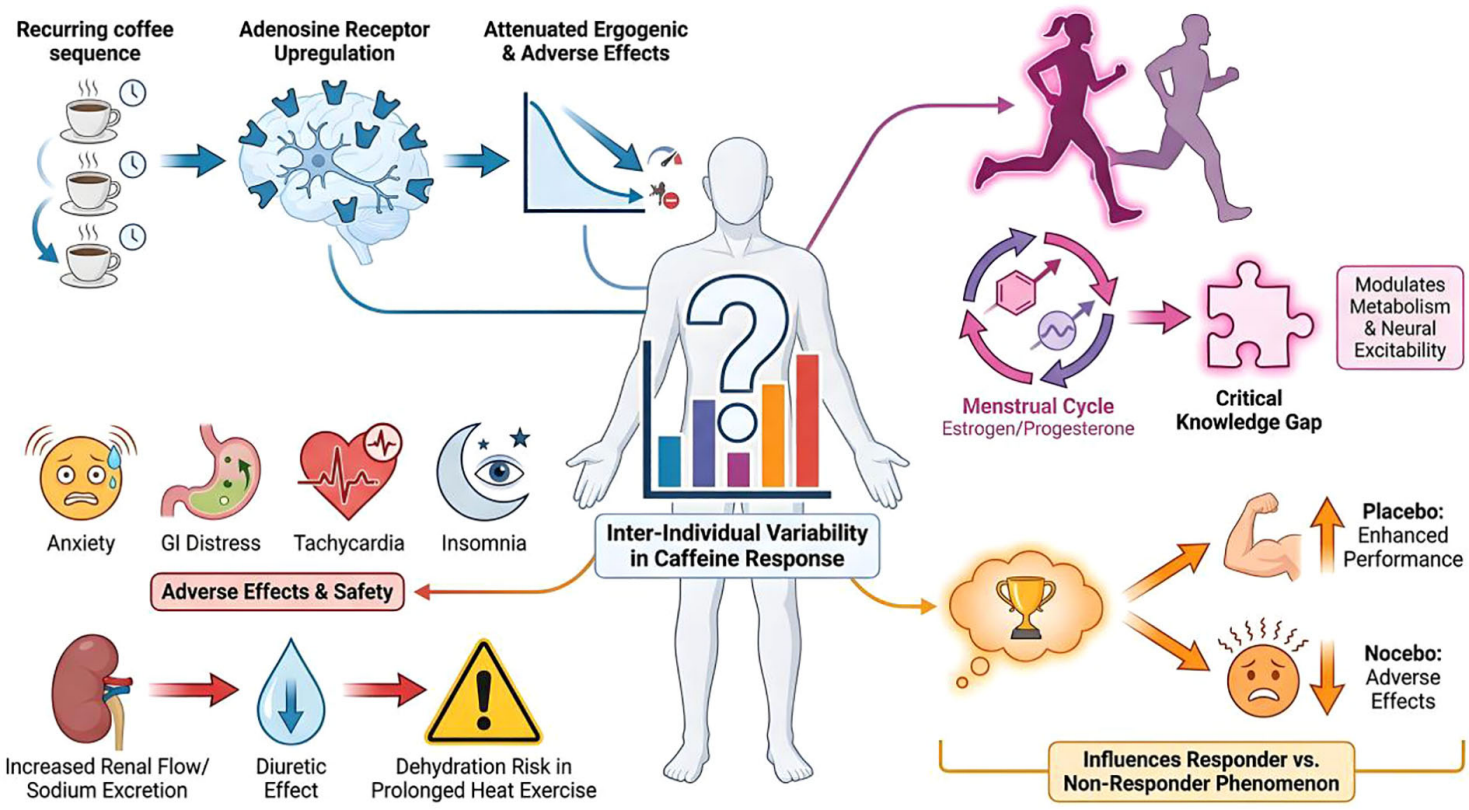
Key determinants of inter-individual variability in caffeine response. The schematic illustrates the primary factors that contribute to the highly variable ergogenic and adverse effects of caffeine between individuals. The left side depicts outcomes of habitual use, including the development of pharmacodynamic tolerance via adenosine receptor upregulation, which attenuates both desired and adverse effects. It also highlights common dose-dependent adverse effects and the diuretic risk. The right side summarizes other modulating factors, such as influences on metabolism and neural excitability, the understudied impact of the menstrual cycle, and the significant role of placebo/nocebo effects. These elements collectively underpin the observed responder versus non-responder phenomenon.

While the preceding sections outline the established framework of caffeine’s mechanisms and key moderators like dose and genetics, a full explanation of the variability observed in both research and practical athletic outcomes requires a critical examination of several additional, often underexplored, factors. These elements are essential for accurately interpreting contradictory findings in the literature, understanding the limits of general recommendations, and advancing truly personalized application strategies (**Figure 4**). This section will therefore scrutinize complex issues such as the implications of habituation and biological sex differences, the powerful confounding role of psychological expectancy, and a balanced appraisal of safety considerations each of which significantly shapes the real-world ergogenic equation.

### 7.1 Habituation, training status, and biological sex

A primary and modifiable source of inter-individual variability is the pharmacodynamic tolerance induced by habitual caffeine use. This adaptation is largely mediated by the compensatory upregulation of central adenosine receptors in response to chronic antagonism by caffeine (Reichert et al., 2022; Lopes et al., 2023). Over time, this upregulation substantially attenuates both the desired ergogenic effects and the common adverse side effects. This physiological tolerance is a critical methodological consideration; it can directly explain null or diminished findings in studies that enroll habitual users without implementing adequate caffeine washout periods prior to testing, thereby confounding the interpretation of caffeine’s true efficacy (Cappelletti et al., 2015; Pickering and Kiely, 2019).

An individual’s training status represents another potential, though less clearly defined, modulator of caffeine’s effects. Training induces profound adaptations in substrate metabolism, neuromuscular efficiency, and neuroendocrine function, all of which are systems that caffeine influences. Consequently, the physiological and performance responses to caffeine may differ between highly trained athletes and recreationally active individuals. For instance, endurance-trained athletes with enhanced baseline fat oxidative capacity might exhibit a blunted metabolic response to caffeine’s lipolytic effects. However, the nature of this interaction whether training potentiates or diminishes caffeine’s efficacy remains poorly defined and is likely highly dependent on the specific exercise task and the athlete’s physiological phenotype.

Biological sex constitutes a critical and persistently under-investigated variable in sports nutrition research, including caffeine supplementation. Female athletes remain markedly underrepresented in this field, creating a significant evidence gap regarding how caffeine interacts with the natural fluctuations in estrogen and progesterone across the menstrual cycle and how this interaction affects exercise performance (Mielgo-Ayuso et al., 2019; Sims et al., 2023). These hormonal fluctuations are known to modulate key physiological parameters such as substrate metabolism, core temperature, and neural excitability, each representing a potential point of interaction with caffeine’s primary and secondary mechanisms. The frequent lack of control, documentation, or phase-specific analysis of the menstrual cycle in research protocols severely limits the generalizability of findings to the female athletic population and undoubtedly contributes to the inconsistency in the broader literature on caffeine and performance. Future research must prioritize the inclusion of female athletes and adopt methodological rigor in accounting for menstrual cycle phase to develop evidence-based, sex-specific guidelines.

### 7.2 Psychological expectancy and the placebo/nocebo effect

Psychological expectancy exerts a significant and often independent influence on ergogenic outcomes, representing a critical confounder in sports supplementation research (Beedie, 2010; Del Coso et al., 2026). The belief that an ingested substance will enhance performance can, in itself, lead to measurable improvements in metrics such as power output, pain tolerance, and reductions in ratings of perceived exertion. Conversely, the anticipation of adverse effects can induce those very symptoms, such as increased anxiety or heart rate palpitations. In both experimental and real-world athletic contexts, participant awareness of having consumed a supplement particularly one as well-known as caffeine activates these potent psychosocial and neurobiological pathways.

Disentangling the specific pharmacological action of caffeine from these expectancy effects is a fundamental methodological challenge, yet it is crucial for accurately interpreting study outcomes and the true efficacy of the compound. This difficulty is most pronounced in trials that are not fully double-blinded or where the distinctive sensory effects of caffeine compromise effective blinding. The interplay between pharmacology and psychology likely underlies a substantial component of the observed responder and non-responder phenomenon. An individual’s pre-existing beliefs about caffeine’s effectiveness, their mindset, and the context of administration can co-determine the outcome as much as the biochemical dose itself. Therefore, the variability in the literature, especially in domains like strength and power where neurological and perceptual factors are primary mediators, may be partly attributed to uncontrolled differences in expectancy between studies and participants. Acknowledging this forces a more nuanced interpretation of caffeine research and underscores the necessity for rigorous blinding procedures and the measurement of subjective beliefs in future studies to isolate the compound’s direct physiological effects.

### 7.3 Safety considerations and potential adverse effects

A balanced and evidence-based perspective on caffeine necessitates a clear acknowledgment of its generally favorable safety profile alongside its dose-dependent risks. Common adverse effects associated with acute intake, particularly at moderate to high doses, include anxiety, gastrointestinal distress, tachycardia, and insomnia (Clark and Landolt, 2017; de Souza et al., 2022; Meamar et al., 2024). The susceptibility to these effects, especially anxiety and sleep disruption, exhibits considerable inter-individual variability, strongly influenced by genetic factors such as polymorphisms in the ADORA2Agene. Therefore, caution or outright contraindication is advised for individuals with predisposing cardiovascular conditions, anxiety disorders, or known hypersensitivity.

The diuretic effect of caffeine, historically a major concern, is mediated by increased renal blood flow and sodium excretion. However, contemporary meta-analytic evidence suggests that in habitual consumers, this effect is mild and transient, and it does not lead to clinically meaningful dehydration or electrolyte imbalance at rest. Nevertheless, in specific athletic contexts, prudent consideration is warranted. For athletes engaged in prolonged exercise in hot and humid environments, or in sports with high baseline fluid loss, the potential diuretic action of caffeine could theoretically accentuate dehydration risk if fluid intake is not consciously and adequately increased. Thus, integrating caffeine use within a disciplined, personalized hydration strategy remains a sensible practice.

Beyond acute effects, the long-term implications of habitual use and the risk of caffeine dependency warrant mention. While true addiction is rare, dependence characterized by tolerance and withdrawal symptoms upon cessation is well-documented, particularly in high-dose chronic users. For athletes, this underscores the importance of periodizing caffeine use and avoiding unnecessary daily high-dose supplementation to maintain its efficacy for competition and mitigate withdrawal disruptions during planned abstinence phases. In summary, while caffeine is safe for most athletes within recommended doses, a strategic approach that respects individual sensitivity, contextual demands, and the potential for dependence is essential to harness its benefits while minimizing adverse outcomes.

## 8 Conclusion and future directions

Caffeine is a well-established ergogenic aid whose effects are mediated through a dose-dependent hierarchy of physiological pathways. Its primary and indispensable mechanism at typical intake levels is the antagonism of central adenosine receptors, which reduces perceived effort, increases arousal, and modulates pain perception. This central effect is the cornerstone of its ergogenic action. Peripheral metabolic consequences, such as enhanced lipolysis, are now understood to play a secondary, supportive role rather than act as a primary driver. The most consistent and robust performance benefits are observed in aerobic endurance exercise, resulting from the synergy of this reduced central neural drive and potential optimization of substrate utilization. In contrast, effects on strength, power, and anaerobic performance are more variable and context-dependent. These benefits, when they occur, are mediated largely through perceptual and neurological modulation rather than direct peripheral metabolic changes, explaining their greater consistency in performance tests involving fatigue or repeated efforts. For skill-based sports requiring high cognitive acuity, caffeine reliably improves reaction time, vigilance, and accuracy via central nervous system stimulation, representing a distinct dimension of ergogenic support. Beyond acute performance, preliminary evidence suggests caffeine may also support post-exercise recovery by potentially accelerating muscle glycogen resynthesis when co-ingested with carbohydrates and by improving muscle perfusion. However, these potential benefits are contingent upon meticulous timing, as caffeine’s potent CNS stimulant effects can severely disrupt sleep architecture, thereby negating any peripheral advantage. Consequently, protecting sleep must be considered a non-negotiable priority over any speculative recovery benefit.

Despite a considerable evidence base, several key areas require targeted research to optimize the personalized application of caffeine. Future studies should prioritize developing and validating sport-specific dosing and timing protocols that account for complex real-world scenarios such as evening competitions, congested schedules, and travel across time zones. The comparative efficacy, pharmacokinetics, and side-effect profiles of different caffeine formulations need clarification. Moreover, the long-term physiological adaptations, the efficacy of washout strategies to reverse tolerance, and the downstream health implications of chronic supplementation in athletes warrant deeper longitudinal investigation. Crucially, there is a pressing need for more research that includes female athletes and controls for menstrual cycle phase, a significant and understudied moderator. Future work must also employ rigorous methodologies to better disentangle the pharmacological effects of caffeine from the powerful influence of psychological expectancy. Ultimately, progress toward true personalized nutrition, habitual intake, training status, and temporal factors will be essential to maximize caffeine’s ergogenic and recovery benefits while minimizing adverse effects, enabling its safe, effective, and individualized application in athletics.
